# Detection of Aspergillus by Nested Assay in Bone Marrow Transplantation (BMT) Patients

**Published:** 2011-04-01

**Authors:** F Teifoori, S H Roudbar Mohammadi, Z Sharifi, H Ghaffari

**Affiliations:** 1 Depattment of Mycology, Tarbiat Modares University of Medical Sciences, Tehran, Iran; 2 Iran Blood Transfusion Organization, Tehran, Iran; 3 Bone Marrow Transplantation Unit, Shariati Hospital, Tehran, Iran

**Keywords:** Invasive aspergillosis, Nested PCR, 18s rRNA, Diagnosis, Bone marrow transplantation

Dear Editor,

The increasing incidence of aspergillosis emphasizes to improve the diagnostic tools.[1] Aspergillus fumigatus is associated with a high rate of mortality among patients receiving Bone Marrow Transplantation (BMT). Diagnosis in the early stages of invasive aspergillosis (IA) is very difficult, as clinical and radiological sings are nonspecific.[2] In this study, we used nested PCR technique to diagnose IA in BMT recipients.

A standard isolate of Aspergillus fumigatus PTCC5009 was grown in potato dextrose broth. DNA extraction of Aspergillus fumigatus, Fusarium oxysporum PTCC5115 and Aspergillus flavus PTCC5004 as control species were done according to established methods.[3][4] Blood samples of 30 patients of Dr. Shariati Hospital as susceptible to IA were obtained. The extraction of fungal DNA was done by DNA purification Kit (Bahar-afshan Company, Tehran, Iran).

The alignment of the three DNA sequences was performed with the program Gene Works (Intelligenetics, Inc.), Selection of the primer sequences was based on a close check for sequences with matching homologies in current DNA databases (Gen Bank). By using a nested PCR technique, two pairs of o primers (AFU5S, AFU5AS and primers AFU7S, AFU7AS) derived from sequences of the A. fumigatus 18s rRNA gene (GenBank No.) were chosen for PCR assays.[5][6] For each PCR mixture, 40 ng of total DNA was used as the template.

The two mentioned primers with the highest sensitivity and species specificity of Aspergillus which amplified a fragment of 404 bp, were followed by AFU5S and AFU5AS which produced an internal fragment of 335 bp. These primer-binding sites are located in the 3' part of the 18S rRNA gene and in variable region V7-V9 (AFU7AS) or V8-V9 (AFU5AS), with no sequence overlap between the primers used in the first and second PCRs to reduce contamination problems. Comparison of clinical characteristics of the patients, and their PCR results showed that IA negative patients had an age>25, WBC count>3000 while positive patients had an age<30 and WBC count=1000-2000.

**Fig. 1:**
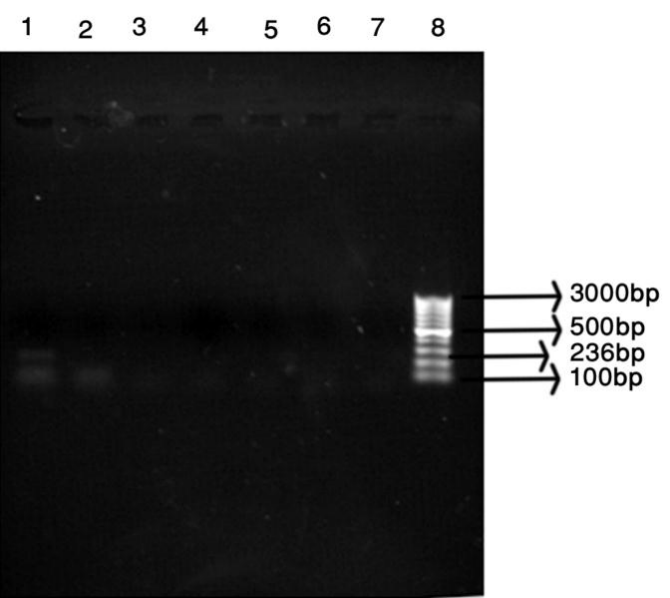
Nested PCR results [1: Positive samples; 8: Marker 50 bp; 2-6: Negative samples; 7: Aspergillus flavus (Negative control)]

Our results showed that out of 30 BMT patients, six patients had positive nested PCR results (20%). So detection of Aspergillus DNA from blood is possible and helpful in diagnosis of IA and can assist in distinguishing this disease from others (Figure 1). Most of the published data used 18S rDNA or 28s rDNA as target DNA but rarely used the internaltranscribed spacers (ITS) regions. The 18S rDNA was selected for several reasons. First, universal fungal primers are based on the conserved regions of 18S rDNA, making it possible to obtain the PCR products from most of the fungi for sequencing. Second, the large number of 18S rDNA sequences in GenBank makes similarity searches convenient and is thus more suitable for finding consensus conserved regions within a group of fungi for developing probes for genus- and group-level detections.[7][8]
